# Caring for End-of-Life Patients and Their Families, During Life, and Mourning, in the COVID-19 Era—The Experience of a Palliative Care Team in Portugal

**DOI:** 10.3389/fpsyt.2021.624665

**Published:** 2021-02-09

**Authors:** Ana Mafalda Carvalheiro, Catarina Faria, Isabel Semeão, Sérgio M. Martinho

**Affiliations:** ^1^Psychiatry and Mental Health Department, Leiria Hospital Center, Leiria, Portugal; ^2^Hospital-Based Palliative Care Team, Leiria Hospital Center, Leiria, Portugal

**Keywords:** end-of-life, mourning, COVID-19, terminally ill patients, prolonged grief, mental health disorders

Throughout history, humankind has survived several epidemics that have caused a high number of deaths and suffering (1). Those events have had profound economic, social, political, cultural, medical, and psychological impacts on humanity (2).

More recently, in December 2019, in Wuhan city in China, a previously unknown coronavirus was identified in humans (3). The new Severe Acute Respiratory Syndrome Coronavirus 2 (SARS-CoV-2) causes a highly contagious and infectious disease, which has been designated Coronavirus Disease 2019 (COVID-19) (4). People with COVID-19 can present a wide variety of symptoms, ranging from mild, or even asymptomatic carriers, to fatal cases, with about one in six infected becoming seriously ill (5). SARS-CoV-2 affects mainly the respiratory tract, resulting in difficulty in breathing, shortness of breath (6), or pneumonia, but can also affect the gastrointestinal, nervous, cardiovascular, dermatologic, or ophthalmic systems (7).

At the time of writing, the number of deaths by coronavirus in the world already exceeds 1,815,518 and continues to increase (8). Experts have warned about the possibility of a new wave during winter 2020 (9), which is already happening in Portugal. Given the high number of deaths, the coronavirus pandemic will leave behind many more to grieve their lives (10). Studies indicate that each COVID-19 death corresponds with approximately nine bereaved people (11).

Faced with this threat in public health, the Directorate-General of Health, which is a division of the Portuguese public administration concerned with public health, prepared the National Preparedness and Response Plan for the Disease Caused by a New Coronavirus, which is a strategic tool to mitigate the impact of the SARS-CoV-2 outbreak (12). This document contains appropriate measures for each phase of the pandemic, focusing on interrupting transmission chains, preventing the spread, reducing the intensity, and decreasing the number of cases (13).

On March 26, 2020, Portugal entered the highest alert and response level against coronavirus—the mitigation phase—and here remains. At that time, one of the measures put in place in Portuguese hospitals advocated that all people with an acute respiratory condition (cough, dyspnea, or breathing difficulty) should be managed as a suspect case (14). However, studies indicate that dyspnea, apart from being one of the hallmark symptoms of COVID-19, can arise from many different underlying conditions (15). Dyspnea is even one of the most frequent and prevalent symptoms in advanced illness of any etiology, whether oncological or non-oncological. It is present in 75% of patients (15), and its prevalence and severity increase with the approach of death—about 70% in the last 6 weeks of life (16). This implies that, even though COVID-19 is not the cause of dyspnea of some patients who resort to the hospital emergency room, including terminally ill patients, they are referred to a dedicated COVID-19 area, tested for SARS-CoV-2, and considered “suspect cases” (15).

According to Portuguese norms, when facing a “suspect case,” family members cannot visit their relatives at the hospital (17, 18). For this reason and on the basis of our experience at the Leiria Hospital Center, we have noticed that most families have tried to keep their terminally ill loved ones at home as long as possible. This situation can lead to caregiver exhaustion and more frequent interventions by the hospital-based palliative care team (HBPCT), with the subsequent overload of the service.

We have also observed that, in most cases, when these patients are brought to the hospital, they are already facing their last days of life and, due to the severity of their clinical condition, end up staying in the COVID-19 area (until they get a negative COVID-19 test result), to later be admitted to a ward.

Regarding patients who remain in the COVID-19 area, difficulties have been identified at various levels. On one hand, the patient has to spend several hours in an area with suspected cases, which substantially increases the risk of contagion. At this point, it is important to mention that this group is, by itself, one of the most vulnerable, having the highest coronavirus death rate (19). On the other hand, HBPCT is not called for symptom management until the patient presents a negative test result for COVID-19. Studies indicate that, in emergency and humanitarian crises, measures to alleviate suffering may be neglected, due to the need to save lives, yet palliative care is also paramount (20). According to several national and international palliative care associations, palliative care should be offered in conjunction with standard treatment for any disease that threatens the continuity of life and should never be associated with omission or exclusion, even during a pandemic (21, 22). For its growing importance, future funding for care and research should not focus only on acute patients and the emergency department but also on palliative care. To add to the previous problems, visits are forbidden in the COVID-19 area (18). Notably, in a normal situation, the law recognizes the right to family support for people with an incurable disease at an advanced stage and in the final state of life (23). However, the COVID-19 pandemic led to an exception regime in which visits are prohibited. All of these constraints lead to increased anxiety and uncertainty in family members and patients.

Regarding patients who are admitted to the wards, the difficulties are not less. Since visits are also suspended here (18), the HBPCT has been trying to circumvent this limitation by allowing virtual visits between the family and the patient, using new technologies, namely, video calls. These calls are organized by a nurse from the HBPCT, and the frequency of contacts is negotiated with the family. In this context, the principles of therapeutic communication and active listening should not be neglected, as they facilitate dialogue with families and patients (20). To facilitate virtual visits, it was necessary to make efforts to obtain financing for the purchase of equipment. Unfortunately, we are aware that there is a small part of the population that will not be able to make use of these services, as they do not have access to the Internet or appropriate electronic equipment. To help patients cope with social isolation, actions that do not jeopardize safety measures, such as providing moments of music or reading, could be attempted (21). In all cases, we face the dilemma between the measures recommended by public health and the understanding of what should be the dignification of the end of life (24). Still regarding inpatients, we have noticed a great difficulty in care responses of the existing palliative care units when facing COVID-19-positive patients with conditions for hospital discharge. According to the technical guidelines issued by the Directorate-General of Health of Portugal, patients can only be admitted to these units after obtaining a negative result (despite being considered clinically cured) (25). This implies that COVID-19-positive patients will receive prolonged support from the HBPCT, with the consequent overload of the team. Regarding the families' reaction to a referral proposal of their loved ones to these units, we have been noticing that many of them accept this possibility only in an extreme situation. This includes situations in which the patient suffers intensely, is agonized, and experiences problems and needs that are difficult to address and that require specific, organized, and interdisciplinary support or situations where caregivers are exhausted. This reaction of families is justified by the fact that patients have to fulfill an isolation period of not <14 days (26), and after this period, visits are restricted to one or two times a week and always after making an appointment. This situation highlights the urgent need for investment in home or community teams, which do not yet exist in our region (26). Currently, there are only a few integrated continuous care teams, which are unable to meet all needs or are less dedicated to the management of palliative patients. When patients are in their last days of life, we offer the opportunity to one of the family members to meet with the patient face to face to say goodbye, using appropriate protective equipment, according to a procedure prepared by the team. While not the ideal situation, this is the only way we can meet the safety conditions. It is important to try to balance ethical procedures, comfort, and patients' life quality with the provided services (20).

In short, despite the fact that many of the terminally ill patients are not infected with the virus, they ended up dying away from family, without being seen by them again, and without being able to say goodbye (27). The natural response of human beings to death, regardless of their culture or religion, is expressed by grief and mourning (28).

Before the pandemic, anticipatory grief would be experienced in the dying process of terminal patients, involving farewell rituals that created opportunities for family communication, solving of unresolved issues, and sharing of good times and opportunities for relational-based conversation on topics such as gratitude, love, and forgiveness (29). In this process, both verbal and nonverbal communication is essential (29). In fact, nonverbal communication seems important in situations where words are insufficient to express what is desired or what cannot be said (29). According to the studies, farewell rituals are promoters of quality of death for patients and quality of life for family members and may favor the resolution of grief (29).

Additionally, during the pandemic mitigation phase and similar to what happened in other countries, the Directorate-General of Health of Portugal created post-death measures (postmortem care, autopsy, and mortuary care), for individuals with suspected or confirmed infection by SARS-CoV-2 (30). As already mentioned, this may include some terminally ill patients with respiratory complaints due to their advanced disease, and not due to the virus. In these cases, as already described by Aguiar et al. (30), the corpses are removed after death and never seen by the family again; identification of the bodies is carried out remotely and through digital photographs of the face; corpses are kept in a bag without the attire usually chosen by the family; caskets are closed and kept closed during the funeral; and funerals and burials are postponed or held remotely or with the presence of a maximum of 10 people (30). These measures have deprived many terminally ill patients and the families of the mourning rituals they have been planning throughout the course of their illness (28). This can make family members feel that they did not say goodbye the way they wished for (31) and that the deceased did not receive the funeral he or she deserved, was victim of negligence, or has undergone inhumane treatment. Notably, in the past, communicating bad news to the family over the telephone was considered an unethical practice (22). In the COVID-19 era, this practice has been important to avoid contact and comply with physical distancing requirements.

What has been happening during the pandemic is that all actions that promote quality mourning, such as religious activities, social support (32–35), and the fulfillment of last wishes, have been limited or postponed indefinitely (22). This may increase the risk of mental health problems, including persistent, severe, and disabling grief, also termed prolonged grief disorder, among both those bereaved due to COVID-19 and those bereaved due to other causes (28, 29, 32–34, 36). Indeed, pioneering quantitative and qualitative studies have suggested that prolonged grief disorder may become a major public health concern following the pandemic (32–34, 36, 37). For this reason, it seems essential to increase accessibility to evidence-based interventions, which include cognitive-behavioral therapy, and to stimulate the development and dissemination of these therapies, in both face-to-face and online formats, if the pandemic is to last for a long period of time (36, 37).

Appropriate aftercare and support may be required to prevent a peak in the prevalence of mental health problems due to loss of a loved one (38). It is important that all healthcare professionals in contact with a mourner are aware of the importance of detecting the risk of prolonged grief and providing appropriate referrals. For bereaved people, there are three levels of intervention which must be activated if necessary, and these include social support or monitoring, counseling and therapy, or specialized intervention (with a grieving support group or individual consultation with a psychologist or psychiatrist) (39). In Portugal, only a few palliative care teams (around 60%) have a formal structured grieving support program to accompany the family/caregivers who have lost a family member (40). This program includes actions and activities such as letters or text messages of condolences, home visits, consultations, telephone calls, family conferences, and group consultations (40). There is no consensus on the ideal time horizon between death and the first contact with the family, but in practice, this occurs between 72 h and 8 weeks (39, 40).

Mourning related to terminal illness is a dimension to reinvent in the current context and should include innovative ways to promote connection with people (before and after death) (41). Mourning rituals and practices must be adapted, using real or virtual alternatives for remembrance and commemoration (42), in order to provide comfort to families, maintaining infection control rules (43). The use of modern technology, such as smartphones or tablets, can help maintain some pseudo-social connectedness, which may minimize the grief experienced because of the separation (44).

However, before death, there are still details that we can improve. The norms established so far refer to the general population and not specifically to end-of-life patients. It is urgent that the institutions create preferential circuits for patients at the end of life, considering their particularities, and flowcharts for asymptomatic terminal patients and with suspected infection, which take into account patient and professional safety.

It is reasonable to expect that, over the years, other epidemics or pandemics will emerge. Therefore, a significant research effort has been made to understand the consequences of grieving during this pandemic, so that we are better prepared for similar future situations. We recommend specific studies into how changes brought about by the pandemic will impact grief of family members of terminally ill patients. This may help improve existing prevention and intervention efforts for severe mental health problems within this population.

## Author Contributions

AC conceived of the presented idea and wrote the manuscript with input from all authors. CF, IS, and SM made the critical revision of the article. All authors provided critical feedback and helped shape the final manuscript.

## Conflict of Interest

The authors declare that the research was conducted in the absence of any commercial or financial relationships that could be construed as a potential conflict of interest.

## References

[1] World Health Organization Fact Sheets: Infectious Diseases. Available online at: https://www.who.int/topics/infectious_diseases/factsheets/en/ (accessed September 24, 2020).

[2] Huremović D Brief history of pandemics (pandemics throughout history). Psychiatry Pandemics. (2019) 7–35. 10.1007/978-3-030-15346-5_2

[3] El Zowalaty ME Järhult JD. From SARS to COVID-19: a previously unknown SARS- related coronavirus (SARS-CoV-2) of pandemic potential infecting humans - Call for a One Health approach. One Health. (2020) 9:100124. 10.1016/j.onehlt.2020.100124 32195311 PMC7075990

[4] Zheng J. SARS-CoV-2: an emerging coronavirus that causes a global threat. Int J Biol Sci. (2020) 16:1678–85. 10.7150/ijbs.45053 32226285 PMC7098030

[5] World Health Organization Q&A on Coronaviruses (COVID-19). Available online at: https://www.who.int/emergencies/diseases/novel-coronavirus-2019/question-and-answers-hub/q-a-detail/q-a-coronaviruses (accessed September 24, 2020).

[6] Harvard Health Publishing COVID-19 Basics. Symptoms, Spread and Other Essential Information About the New Coronavirus and COVID-19. Available online at: https://www.health.harvard.edu/diseases-and-conditions/covid-19-basics (accessed September 24, 2020).

[7] Baj J Karakuła-Juchnowicz H Teresiński G Buszewicz G Ciesielka M Sitarz E. COVID-19: specific and non-specific clinical manifestations and symptoms: the current state of knowledge. J Clin Med. (2020) 9:1753. 10.3390/jcm9061753 32516940 PMC7356953

[8] European Centre for Disease Prevention and Control COVID-19 Situation Update Worldwide, as of 24 September 2020. Available online at: https://www.ecdc.europa.eu/en/geographical-distribution-2019-ncov-cases (accessed December 31, 2020).

[9] Mortazavi SS Assari S Alimohamadi A Rafiee M Shati M. Fear, loss, social isolation, and incomplete grief due to COVID-19: a recipe for a psychiatric pandemic. Basic Clin Neurosci. (2020) 11:225–32. 10.32598/bcn.11.covid19.2549.1 32855782 PMC7368098

[10] Gesi C Carmassi C Cerveri G Carpita B Cremone IM Dell'Osso L. Complicated grief: what to expect after the coronavirus pandemic. Front Psychiatry. (2020) 11:489. 10.3389/fpsyt.2020.00489 32574243 PMC7264152

[11] Verdery AM Smith-Greenaway E Margolis R Daw J. Tracking the reach of COVID-19 kin loss with a bereavement multiplier applied to the United States. Proc Natl Acad Sci U.S.A. (2020) 117:17695–701. 10.1073/pnas.2007476117 32651279 PMC7395491

[12] Direção-Geral da Saúde Plano Nacional de Preparação e Resposta à Doença por novo coronavirus (COVID-19). Available online at: https://covid19.min-saude.pt/wp-content/uploads/2020/03/Plano-de-Conting%C3%AAncia-Novo-Coronavirus_Covid-19.pdf (accessed September 24, 2020).

[13] European Centre for Disease Prevention and Control Outbreak of Novel Coronavirus Disease 2019 (COVID-19): Increased Transmission Globally - Fifth Update. Available online at: https://www.ecdc.europa.eu/sites/default/files/documents/RRA-outbreak-novel-coronavirus-disease-2019-increase-transmission-globally-COVID-19.pdf (accessed September 24, 2020).

[14] Direção-Geral da Saúde Norma n° 004/2020 de 23/03/2020. COVID-19: FASE DE MITIGAÇÃO. Abordagem do Doente com Suspeita ou Infeção por SARS-CoV-2. Available online at: https://www.dgs.pt/normas-orientacoes-e-informacoes/normas-e-circulares-normativas/norma-n-0042020-de-23032020-pdf.aspx (accessed September 24, 2020).

[15] Fraser Health Authority Hospice Palliative Care Program. Symptom Guidelines. Dyspnea Available online at: https://www.fraserhealth.ca/-/media/Project/FraserHealth/FraserHealth/Health-Professionals/Professionals-Resources/Hospice-palliative-care/Dyspnea.pdf (accessed September 24, 2020).

[16] Reuben DB Mor V Dyspnea in terminally ill cancer patients. Chest. (1986) 89:234–6. 10.1378/chest.89.2.234 3943383

[17] Direção-Geral da Saúde Norma n° 002/2020 de 25/01/2020. Infeção Pelo Novo Coronaví*rus (2019-nCoV)* Available online at: https://www.dgs.pt/directrizes-da-dgs/orientacoes-e-circulares-informativas/orientacao-n-0022020-de-25012020-pdf.aspx (accessed September 24, 2020).

[18] Centro Hospitalar de Leiria Notícias e eventos. CHL Mantém a Suspensão da Entrada de Acompanhante/Cuidador/Visitas. Available online at: http://www.chleiria.pt/comunicacao-social/noticias-eventos/-/chl-mantem-a-suspensao-da-entrada-de-acompanhantecuidadorvisitas-589/ (accessed November 27, 2020).

[19] Wang SSY Teo WZY Yee CW Chai YW. Pursuing a good death in the time of COVID-19. J Palliat Med. (2020) 23:754–55. 10.1089/jpm.2020.0198 32311289

[20] Florêncio RS Cestari VRF Souza LC Flor AC Nogueira VP Moreira TMM. Cuidados paliativos no contexto da pandemia de COVID-19: desafios e contribuições. Acta Paul Enferm. (2020) 33:eAPE20200188. 10.37689/acta-ape/2020AO0188632997068

[21] Associação Portuguesa de Cuidados Paliativos Cuidados Paliativos. O Que São? Available online at: https://www.apcp.com.pt/cuidados-paliativos/o-que-sao.html (accessed November 27, 2020).

[22] Artmed Panamericana Editora Ltda Cuidados Paliativos No Contexto da Covid-19: Como Proceder. Available online at: https://secad.artmed.com.br/blog/coronavirus/cuidados-paliativos-na-covid-19/ (accessed November 27, 2020).

[23] Diário da República Electrónico Direitos e Deveres do Utente dos Serviços de Saúde. Lei n.° 15/2014 - Diário da República n.° 57/2014, Série I de 2014-03-21. Available online at: https://dre.pt/home/-/dre/571943/details/maximized (accessed November 27, 2020).

[24] Rocha C Oliveira HM Cuidados paliativos na pandemia COVID-19. Med Intern. (2020) 27:44–7. Available online at: 10.24950/rspmi/COVID19/C.Rocha/H.M.Oliveira/ULSM/S/2020

[25] Direção-Geral da Saúde Orientação n° 009/2020 de 11/03/2020. Atualização: 23/07/2020. COVID-19: Fase de Mitigação Available online at: https://www.dgs.pt/directrizes-da-dgs/orientacoes-e-circulares-informativas/orientacao-n-0092020-de-11032020-pdf.aspx (accessed November 27, 2020).

[26] Serviço Nacional de Saúde Plano Estratégico Para o Desenvolvimento dos Cuidados Paliativos. Biénio 2019–2020. Available online at: https://www.sns.gov.pt/wp-content/uploads/2019/04/PEDCP-2019-2020-versao-final-10.02.2019.pdf (accessed November 27, 2020).

[27] Wallace CL Wladkowski SP Gibson A White P. Grief during the COVID-19 pandemic: considerations for palliative care providers. J Pain Symptom Manage. (2020) 60:e70–e6. 10.1016/j.jpainsymman.2020.04.012 32298748 PMC7153515

[28] Farahmandnia B Hamdanieh L Aghababaeian H COVID-19 and unfinished mourning. Prehosp Disas Med. (2020) 35:464 10.1017/S1049023X20000631 PMC7251281 32393415

[29] Crepaldi MA Schmidt B Noal DS Bolze SDA Gabarra LM 2020 Terminalidade, morte e luto na pandemia de COVID-19: demandas psicológicas emergentes e implicações práticas. Estudos Psicol (Campinas). (2020) 37:e200090 10.1590/1982-0275202037e200090

[30] Aguiar A Pinto M Duarte R. Grief and mourning during the COVID-19 pandemic in Portugal. Acta Med Port. (2020) 33:543–5. 10.20344/amp.14345 32662415

[31] Burrell A Selman LE. How do funeral practices impact bereaved relatives' mental health, grief and bereavement? A mixed methods review with implications for COVID-19. Omega (Westport). (2020) 1–39. 10.1177/0030222820941296 32640878 PMC9185109

[32] Stroebe M Schut H. Bereavement in times of COVID-19: a review and theoretical framework. OMEGA J Death Dying. (2020) 82:500–22. 10.1177/0030222820966928 33086903 PMC7756059

[33] Eisma MC Tamminga A. Grief before and during the COVID-19 pandemic: multiple group comparisons. J Pain Symptom Manage. (2020) 60:e1–e4. 10.1016/j.jpainsymman.2020.10.004 33065207 PMC7553097

[34] Hamid W Jahangir MS. Dying, death and mourning amid COVID-19 pandemic in Kashmir: a qualitative study. OMEGA J Death Dying. (2020). 10.1177/0030222820953708 32862778

[35] Breen LJ Grief loss and the COVID-19 pandemic. Aust J Gen Pract. (2020) 49 10.31128/AJGP-COVID-20 32416647

[36] Eisma MC Tamminga A Smid GE Boelen PA. Acute grief after deaths due to COVID-19, natural causes and unnatural causes: an empirical comparison. J Affect Disord. (2021) 278:54–6. 10.1016/j.jad.2020.09.049 32950843 PMC7487144

[37] Eisma MC Boelen PA Lenferink LIM. Prolonged grief disorder following the Coronavirus (COVID-19) pandemic. Psychiatry Res. (2020) 288:113031. 10.1016/j.psychres.2020.113031 32360895 PMC7194880

[38] Yahya AS Khawaja S. Bereavement and grief during the COVID-19 pandemic. Prim Care Companion CNS Disord. (2020) 22:20com02661. 10.4088/PCC.20com02661 32589826

[39] Ordem dos Enfermeiros Apoio ao Luto em Familiares de Clientes em Cuidados Paliativos. Available online at: https://www.ordemenfermeiros.pt/arquivo/projectos/Documents/Projetos_Melhoria_Qualidade_Cuidados_Enfermagem/HospitalLuz_ApoioLutoFamiliaresClientesCuidadosPaliativos.pdf (accessed November 27, 2020).

[40] Católica Instituto de Ciências da Saúde Observatório Português dos Cuidados Paliativos. Relatório de Outono (2018). Available online at: https://www.apcp.com.pt/uploads/opcp_projeto_3_relatorio_2018_vf-1.pdf (accessed November 27, 2020).

[41] Helton G Wolfe J Snaman JM. Definitely mixed feelings: the effect of COVID-19 on bereavement in parents of children who died of cancer. J Pain Symptom Manag. (2020) 60:e15–e20. 10.1016/j.jpainsymman.2020.08.035 32889042 PMC7467087

[42] Münch U Müller H Deffner T von Schmude A Kern M Kiepke-Ziemes S Empfehlungen zur Unterstützung von belasteten, schwerstkranken, sterbenden und trauernden Menschen in der Corona-Pandemie aus palliativmedizinischer Perspektive. Empfehlungen der Deutschen Gesellschaft für Palliativmedizin (DGP), der Deutschen Interdisziplinären Vereinigung für Intensiv- und Notfallmedizin (DIVI), des Bundesverbands Trauerbegleitung (BVT), der Arbeitsgemeinschaft für Psychoonkologie in der Deutschen Krebsgesellschaft, der Deutschen Vereinigung für Soziale Arbeit im Gesundheitswesen (DVSG) und der Deutschen Gesellschaft für Systemische Therapie, Beratung und Familientherapie (DGSF). Schmerz. (2020) 34:303–13. 10.1007/s00482-020-00483-9 32488422 PMC7265165

[43] Mayland CR Harding AJE Preston N Payne S. Supporting adults bereaved through COVID-19: a rapid review of the impact of previous pandemics on grief and bereavement. J Pain Symptom Manage. (2020) 60:e33–e9. 10.1016/j.jpainsymman.2020.05.012 32416233 PMC7228694

[44] Singer J Spiegel JA Papa A. Preloss grief in family members of COVID-19 patients: recommendations for clinicians and researchers. Psychol Trauma. (2020) 12:S90–S3. 10.1037/tra0000876 32478543

